# Supporting gut health with medicinal cannabis in people with advanced cancer: potential benefits and challenges

**DOI:** 10.1038/s41416-023-02466-w

**Published:** 2023-10-26

**Authors:** Hannah R. Wardill, Luke T. Wooley, Olivia M. Bellas, Katrina Cao, Courtney B. Cross, Madele van Dyk, Ganessan Kichenadasse, Joanne M. Bowen, Andrew C. W. Zannettino, Sepehr Shakib, Gregory B. Crawford, Jaroslav Boublik, Mellar M. Davis, Scott D. Smid, Timothy J. Price

**Affiliations:** 1 The School of Biomedicine, Faculty of Health and Medical Sciences, The University of Adelaide, Adelaide, SA Australia; 2 Supportive Oncology Research Group, Precision Cancer Medicine Theme, South Australian Health and Medical Research Institute (SAHMRI), Adelaide, SA Australia; 3 School of Public Health, Faculty of Health and Medical Sciences, The University of Adelaide, Adelaide, SA Australia; 4 Flinders Centre for Innovation in Cancer, Flinders Medical Centre/Flinders University, SA Health, Adelaide, SA Australia; 5 Northern Adelaide Local Health Network South Australia, SA Health, Adelaide, SA Australia; 6 Adelaide Medical School, Faculty of Health and Medical Sciences, The University of Adelaide, Adelaide, SA Australia; 7 LeafCann Group Pty Ltd, Coolum Beach, QLD Australia; 8 The Geisinger Commonwealth School of Medicine, Scranton, PA USA; 9 Queen Elizabeth Hospital, Adelaide, SA Australia

**Keywords:** Chemotherapy, Digestive signs and symptoms

## Abstract

The side effects of cancer therapy continue to cause significant health and cost burden to the patient, their friends and family, and governments. A major barrier in the way in which these side effects are managed is the highly siloed mentality that results in a fragmented approach to symptom control. Increasingly, it is appreciated that many symptoms are manifestations of common underlying pathobiology, with changes in the gastrointestinal environment a key driver for many symptom sequelae. Breakdown of the mucosal barrier (mucositis) is a common and early side effect of many anti-cancer agents, known to contribute (in part) to a range of highly burdensome symptoms such as diarrhoea, nausea, vomiting, infection, malnutrition, fatigue, depression, and insomnia. Here, we outline a rationale for how, based on its already documented effects on the gastrointestinal microenvironment, medicinal cannabis could be used to control mucositis and prevent the constellation of symptoms with which it is associated. We will provide a brief update on the current state of evidence on medicinal cannabis in cancer care and outline the potential benefits (and challenges) of using medicinal cannabis during active cancer therapy.

## Introduction

Despite the excitement that surrounds newer, more targeted agents, the reality for most people with advanced cancer is that chemotherapy will be used, and it will cause a degree of collateral damage to healthy tissues [1]. Clinically, this damage presents as a broad variety of diverse, individualised, and highly dynamic symptoms and side effects. Rarely do these side effects occur in isolation; instead, they present as clusters of related symptoms that are united by common underlying mechanisms, as well as physical and psychosocial/behavioural determinants [2]. With increased accessibility of “big”, real-world data, these symptom clusters have been documented and characterised with greater precision [3]. This has prompted new initiatives to identify early drivers of chronic treatment-related morbidity, with the goal of halting the self-perpetuating nature of inter-related symptom clusters.

Of the many documented side effects of chemotherapy, the breakdown of the mucosal barrier of the gastrointestinal tract (“mucositis”) is one of the earliest and most common. Mucositis is initiated by rapid and extensive DNA damage in highly proliferative stem cells throughout the gastrointestinal mucosa [4]. The resulting apoptosis and inflammation degrades the mucosa, leading to the formation of ulcerative lesions in the mouth, oesophagus, intestines and rectum which severely impair functional capacity. This dysfunction can lead to taste changes, dysphagia, pain and malabsorption; each of which drive anorexia, malnutrition and dehydration [5]. On a cellular level, these breaches in the protective mucosa create an inhospitable environment for resident gut bacteria, leading to loss of commensal species and their protective metabolites including short-chain fatty acids (SCFAs). These changes further weaken the mucosal barrier and permit unrestricted communication between the underlying immune system and luminal compounds (e.g., danger signals). This results in profound local and systemic inflammation which leads to numerous extraintestinal consequences such as fever (“febrile mucositis”) [6], cognitive impairment [7, 8] and fatigue [9]. As such, the destructive changes in the gastrointestinal microenvironment position mucositis as a catalyst for a range of secondary complications, and a key player in a range of symptom clusters.

Despite the impact of mucositis on patients and the healthcare system, it remains without effective intervention, and its range of secondary symptoms/consequences are managed reactively and in isolation [10]. Given the body of evidence that now suggests many symptoms and treatment consequences may be influenced by mucositis, there is an opportunity to control mucositis to mitigate the constellation of impactful symptoms with which it is associated. This review aims to outline a rationale for how, based on its already documented effects on the gastrointestinal microenvironment, medicinal cannabis could be used to control mucositis and prevent its associated symptom cluster. We will provide a brief update on the current state of evidence on medicinal cannabis in cancer care and outline the potential benefits (and challenges) of using medicinal cannabis during active cancer therapy.

## Medicinal cannabis: illicit drug, plant, or medicine?

Cannabis has been used medicinally for over 3000 years, primarily for its analgesic properties. The predominant phytocannabinoids in cannabis by amount are Δ^9^-tetrahydrocannabinol (Δ^9^-THC or THC) and cannabidiol (CBD), with mainstay medicinal cannabis (MC) preparations containing either or both of these compounds in varying ratios as the active ingredients, either as isolates or whole extracts. However, there remains a vast phytochemical complexity to cannabis aside from just THC and CBD, whereby whole extracts may contain over one hundred different minor phytocannabinoids and terpenes, all of which may vary in their relative expression across a number of cannabis chemotypes and displaying variable retention via different extraction processes [11, 12]. Many of these compounds have been shown to exhibit selective bioactivities that may interact with the efficacy of THC and CBD in MC preparations and may be considerations particularly where full-spectrum botanical extracts are concerned, oft described through the popular term ‘entourage effect’ applied to medicinal cannabis [13, 14]. However, their contribution to the efficacy of most conventional MC formulations where THC and CBD predominate may only be marginal, with a paucity of investigative studies on these cannabis phytochemicals compared to THC and CBD.

Today, over 40 countries have legalised MC, with different access pathways depending on jurisdictional legislation [15]. Despite significant variation across jurisdictions, the medicinal use of cannabis is guided by the formulation (oil, sprays, tablets and flowers). Typically, MC requires prescription for a pre-defined indication or with clear clinical justification for why a certain condition may respond positively to MC. This ambiguity often results in lengthy and administratively burdensome reporting requirements; hence, MC use continues to be a challenging medico-legal entity. The combination of its often high cost, clinician hesitancy and logistic difficulties for supply compared to relative ease of access in the community, is a major driver of why people continue to self-medicate with uncontrolled and non-standardised cannabis products.

Canada was one of the first countries to introduce a MC access programme in 1999. Between 2015 and 2019, the number of registered MC patients in Canada increased from 40,000 to nearly 400,000, an increase attributed to several policy changes that have gradually broadened access to a variety of medicinal cannabis formulations. In December 2015, the first cannabis oil product was launched in the Canadian market and in 2016, the Access to Cannabis for Medicinal Purposes Regulations allowed patients to grow cannabis for personal use [16].

The United States was another early adopter of medicinal cannabis, starting with state-level legalisation in California in 1996. Now, as of February 2023, medicinal cannabis has been legalised in 37 states, 3 territories, and the District of Columbia [17]. In February 2019, Thailand became the first and only nation in Southeast Asia to legalise medicinal cannabis, offering three different categories of cannabis-based products: medicinal-grade cannabis-based medicines, Thai traditional medicine products that contain cannabis as the active ingredient, and folk medicine products prepared by registered folk healers [18].

In 2016, cannabis was rescheduled in Australia to enable access for medicinal purposes. Currently, the majority of cannabis products are classified as “unapproved” therapeutic goods, with two exceptions: Sativex (nabiximols), an oromucosal spray containing equivalent amounts of THC and CBD, and Epidyolex (also known as Epidiolex), a CBD solution with a concentration of 100 mg/mL (Therapeutic Goods Administration, 2020a). The situation in the UK is similar; even though medicinal cannabis was legalised in 2018, it remains challenging for patients to obtain access, with only a limited number of National Health Service prescriptions issued to date [19]. Medicinal products that meet safety, quality and efficacy standards are registered on the Australian Register of Therapeutic Goods (ARTG), however additional pathways such as Special Access Schemes (SAS) and an Authorised Prescriber (AP) scheme facilitate patient access to “unapproved” therapeutic goods, including many other types of MC products currently. This scheme is being increasingly adopted in Australia, with the MC Therapeutic Goods Administration (TGA) dashboard showing an increase in AP applications and a levelling off of SAS-B approvals in the last 2 years.

## The “endocannabinoidome” and its relevance to chemotherapy symptoms and side effects

The endocannabinoidome or, in its broader sense, the endocannabinoid system (ECS), is an endogenous network of receptors, enzymes, transporters and ligands, that has largely been recognised for its role in regulating of neurotransmitter release [20]. However, this original concept of the ECS is gradually being replaced by an increasingly sophisticated and complex network capable of regulating numerous biological pathways and functions across a range of organ systems. The interactions that exist within the ECS are critical to central nervous system development, synaptic plasticity and the homeostatic maintenance of cognitive, behavioural, emotional, developmental and physiological processes [20, 21]. These diverse mechanisms are mediated by endogenous cannabinoids which are produced on demand, both physiologically and patho-physiologically, from membrane lipids and are metabolised by fatty acid amide hydrolase (FAAH) and monoacylglycerol lipase (MAGL), respectively [22]. They include the main known endocannabinoids, N-arachidonoylethanolamine (AEA) or anandamide and 2-arachidonoylglycerol (2-AG) produced by phospholipid precursors through activity-dependent activation of specific phospholipase enzymes; N-acyl-phosphatidylethanolamine-selective phosphodiesterase and phospholipase C subsequent production of diacylglycerol via diacylglycerol lipase, respectively [20]. The broadly termed acylethanolamines and acylglycerols interact to a variable extent with cannabinoid receptors including the well-described CB1 and CB2 receptors (CNR1/CNR2) as well as other G-protein-coupled receptors such as GPR55, GPR18, GPR3, GPR6, GPR12, transient receptor potential channels such as TRP vanilloids TRPV1 to TRPV4, TRP ankyrin TRPA1, TRP M member TRPM8 and peroxisome proliferator-activated receptors such as PPAR2, and PPARγ [23]. Although there are diverse receptors with which endocannabinoids interact, CNR1 and CNR2 are functionally characterised both physiologically and with respect to ECS dysfunction.

**Fig. 1:**
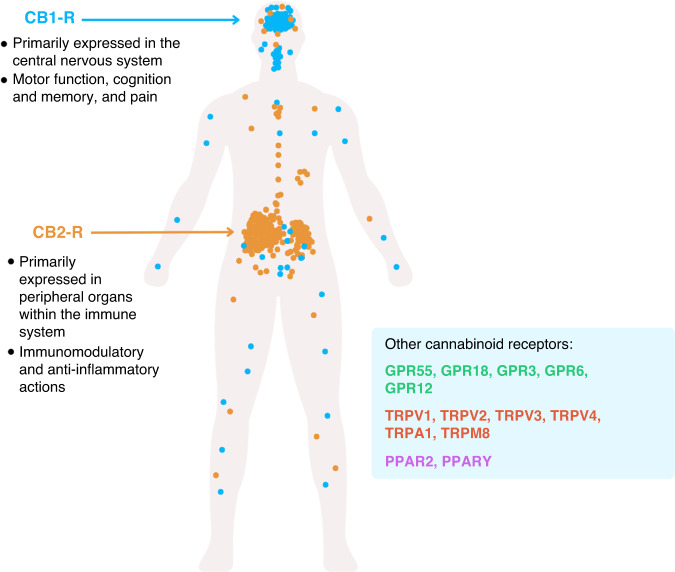
Simplified scheme representing the expression pattern of the main cannabinoid receptors, CNR1 and CNR2. In addition to CNR1 and CNR2, including GPCRs (GPR18, GPR55, GPR3, GPR6, and GPR12), the receptor potential (TRP) channels (TRPV1, TRPV2, TRPV3, TRPV4, TRPA1 and TRPM8) and peroxisome proliferator-activated receptors (PPAR2 and PPARγ).

CNR1 is a G-protein-coupled receptor that is highly abundant within the peripheral and central nervous system, largely present on axon terminals and pre-terminal axon segments [24] (Fig. 1). It is highly abundant in medium spiny neurons in both the dorsal and ventral striatum, and is particularly high on the direct pathway axons as they enter the globus pallidus heading towards the substantia nigra [25]. However, the expression of CNR1 is increasingly diverse, with a range of immune cells (including glia) now understood to express CNR1 [26–28] as well as the more immunologically abundant, CNR2. Indeed, there remains debate about the expression of CNR2 which has historically been considered to be mainly expressed in the periphery [29]. Although emerging evidence suggests it is expressed on sensory nerve terminals and microglia in the brain [30].

Although not the only components of cannabis, the most commonly studied and clinically utilised components, THC and CBD, are highly lipid soluble and have a poor bioavailability when inhaled (10–35%) or orally ingested and 11–45% [31–33]. When THC is consumed by inhalation or mucosal sprays, it is absorbed through the lungs into the bloodstream and concentrations in the plasma will typically peak in less than 10 min [31] and when it is orally congested, concentrations may peak at ~2 h. THC is distributed into well vascularises organs as well as the brain. THC and CBD are highly protein-bound and has a half-life of 25–36 and 18–32 h, respectively [31, 34], with some of the metabolites (THCCOOH) having a very long half-life up to 52 h [35]. Chronic users have much longer half-lives.

THC undergoes extensive first-pass metabolism in the liver by cytochrome P450 (CYP 450) isozymes; these enzymes are also responsible for the metabolism of many anti-cancer drugs and several other commonly used co-medication and are known to cause large inter-individual variability in plasma concentrations for majority of clinically used medications [36]. Therefore, it is particularly important to investigate any potential drug–drug interactions to avoid toxicity or therapeutic failure for any of the medications. THC primarily is metabolised via oxidation by CYP2C9, CYP2C19 and CYP3A4 into several metabolites with 11‐hydroxy‐THC (11‐OH‐THC) and 11‐carboxy‐THC (11‐COOH‐THC) being the most abundant [34, 37]. 11-OH-THC is metabolised by the UGT1A9 and UGT1A10 enzymes and 11-COOH-THC is metabolised mostly by the UGT1A3 and UGT2B7enzyme [34, 35]. CBD is also metabolised in the liver via mostly CYP3A4, CYP2C19 and CYP2C9 and other CYPs to a lesser degree via hydroxylation to form metabolites, 7-OH-CBD and 7-COOH-CBD, which is then undergoes glucuronidation by UGT1A9 and UGT2B7 [31, 34]. The remaining THC, CBD and metabolites can be taken up by fat tissue before it is redistributed into the circulation. More than 65% of THC and CBD will be excreted in faeces and ~20% in the urine [35, 37]. Metabolites excreted in urine have been observed to vary up to fivefold between individuals when drug administration was controlled, demonstrating the variability in metabolism [35].

Inter-individual differences in pharmacogenomics, pharmacokinetics and pharmacodynamics may explain contradictory outcomes from previous studies and accounting for such differences will provide an opportunity for personalised medicine where efficacy can be maximised, and toxicity minimised for various conditions or diseases [38].

Reflecting the breadth of cells upon which ECS receptors are expressed, the ECS regulates a number of critical functions that are well-known to contribute to the side effects of chemotherapy. Most notable is its psychotropic properties, modulating mood, anxiety, cognition, appetite, sleep and pain [39, 40]; all of which are well-documented to be negatively impacted by chemotherapy [1]. Peripheral CB1 expression is also implicated in gastrointestinal inflammation, mucosal defences and gastric motility [41–44], and thus by extension diarrhoea and constipation, due to its expression on presynaptic cell of sympathetic motor neurons innervating visceral organs leading to reduced noradrenalin release [45]. Further to this, given the immunomodulatory capacity of the ECS, its ability to influence numerous symptoms and side effects of chemotherapy of which many are underpinned by aberrant inflammation, is vast. It is for these reasons that medicinal cannabis, and strategies to augment the ECS, have gained considerable momentum for their potential benefits in people with cancer.

## Medicinal cannabis use in cancer care: what is the evidence?

Cannabis use in people with cancer is not uncommon, although it remains difficult to determine exact prevalence due to heterogeneous results published across numerous studies with varied designs. In a study published in 2018, 43% of respondents (at a Canadian cancer centre) reported using “illicit” cannabis for a variety of symptoms and side effects of their treatment [46]. This is similar to a large population-based analysis from 2005 to 2014 in the US, which showed 40.3% of the 826 respondents with cancer having used cannabis in the last 12 months [47]. However, in a larger survey of more than 200,000 people, results indicated that less than 10% of people were using cannabis [48]. Irrespective of self-reported cannabis use, there is a high degree of interest in its potential benefits during cancer care, with 80% of healthcare professionals reporting that they have engaged in conversations with their patients about cannabis [49]. Unfortunately, less than 30% feel equipped to guide their patients citing a lack of clear evidence on its safety and efficacy [49]. This uncertainty undoubtedly stems from the inadequate and highly variable evidence base for cannabis in cancer care, which is dominated by largely observational studies that are subject to inherent biases, powerful placebo effects and diverse confounders, leading to a high rate of false positives [50–52]. Similarly, of the limited number of randomised control trials, few are considered high quality and they remain near impossible to compare/synthesise due to inherent differences in design, outcome measures and cannabis products/doses/delivery/formulations used [50]. This has prevented replication and meta-analyses, and the resulting evidence base is therefore inconsistent and largely uninformative.

Although challenging to compare studies, a large number of systematic reviews have been conducted in an attempt to synthesise data and determine its efficacy in symptom control. Notably, there have been few that have been able to perform meta-analysis, reflecting the heterogeneity of available data. The most recent review of cannabis in cancer care reviewed 42 studies (19 randomised, 23 non-randomised), focused on people with cancer receiving palliative care [53]. Among these studies, pain was the most commonly investigated symptom, with highly variable effects reported across the studies. This aligns with a recent systematic review and meta-analysis which investigated the effect of cannabis for pain management in people with cancer, which was unable to form any conclusive recommendation [54]. Accordingly, recent guidelines from the Multinational Association for Supportive Care in Cancer (MASCC) do not recommend the use of cannabinoids for cancer pain, although, it is unclear how this relates to *chemotherapy-induced* pain which is diverse in its origins [52].

While pain has dominated the landscape for cannabis research in cancer care, emerging evidence exists for its role in chemotherapy-induced nausea and vomiting (CINV), anorexia, cachexia, sleep disturbance and psychological symptoms (depression/anxiety). Doppen and colleagues reviewed the evidence for CINV, reporting generally positive effects across multiple studies using various assessment tools [53]. This is consistent with recommendations from MASCC, which show THC and nabilone are both effective in controlling CINV, however, no more effective than current antiemetic medications [55]. Despite these positive findings, MASCC was unable to form any guideline due to insufficient, high-quality evidence. This is echoed by the American Society for Clinical Oncology (ASCO) who question the quality of current evidence [56]. Despite the lack of clinical recommendation from MASCC and ASCO, it appears that there is discordance between published clinical trial data and anecdotal reports of patient preferences, which tend to favour cannabis over existing antiemetic strategies, even when adverse effects were higher [57, 58].

MASCC maintains a similar stance with respect to anorexia and cachexia, with limited evidence available to inform relevant guidelines. Doppen et al. reviewed 15 studies for cannabis and appetite, with both objective and self-reported benefits reported for nabinol and Marinol, however, several studies reported no or inconsistent effects [53]. Given the heterogeneity in data and approaches, it remains difficult to draw robust conclusions despite the popularised effects of cannabis on appetite stimulation. Similarly, for psychological effects (e.g., on sleep, anxiety, depression), MASCC were unable to make any recommendations with most studies investigating these outcomes as secondary analyses with inconsistent data across studies [51].

While the systematic review by Doppen et al. has provided insight on the current state of evidence regarding cannabis use in cancer care, it has been scrutinised for its methodology and over-simplification of data as “positive” and “negative” effects based on the null hypothesis significance test [50, 53]. As cautioned by Davis and Soni (2022), our approach to cannabis research should be guided by effect sizes that are deemed clinically significant, rather than statistically significant [50]. Furthermore, they highlight the need to be more holistic in our assessment of cannabis for symptom management, avoiding excessively large studies designed with highly restrictive outcomes guided by narrow-minded criteria [50]. With this in mind, and the growing appreciation for symptom clusters in people undergoing chemotherapy, there is a clear rationale to prioritise trial designs that address clusters of related symptoms, rather than single symptoms, to deliver meaningful impacts to the participant’s physical or psychosocial well-being. Critical to these approaches is the inclusion of consumers in cannabis research, to ensure research methodologies are informed by, and consistent with, consumers behaviours and preferences. In line with ensuring consumer engagement, trials should include relevant patient reported outcome measures (PROMs) to ultimately determine if cannabis has a meaningful impact on people with cancer.

## Gastrointestinal effects of cannabis: can they be adapted to control mucositis?

Cannabinoids, including both CBD and THC, are increasingly documented for their capacity to modulate gastrointestinal function, owing to the immense control that the ECS has on gastrointestinal homeostasis. Both CNR1 and CNR2 are present in the gastrointestinal tract, largely on enteric nerves and the epithelium, but also on enteroendocrine cells and immune cells [42]. This network of ECS receptors controls gastric motility, and as such, it is now understood that variants in the genes encoding for CNR1 are implicated in diseases characterised by altered motility including irritable bowel syndrome (IBS), particularly diarrhoea-predominant [59]. Both CNR1 and CNR2 are expressed in the gut at medium to high degrees, respectively. Accordingly, it has been shown that agonists of the cannabinoid receptors (CNR1 and CNR2) [60, 61], as well as targeting endocannabinoid degradation [62], minimises experimental colitis and associated visceral hypersensitivity [63]. Furthermore, preclinical investigations have shown that potent agonists of CNR1 (without central nervous system effects) and CNR2 have been shown to control increased gastrointestinal motility caused by stress [64] and inflammation [44]. Similarly, inhibition of anti-diacylglycerol lipase (DAGL) and fatty acid amide hydrolase (FAAH)—two enzymes critical in endocannabinoid metabolism—has been shown to normalise transit time in the context of opioid-induced constipation [65]. Of particular interest to changes in the gastrointestinal microenvironment associated with mucositis is the ability of CNR2 activation [66] and FAAH inhibition [67] to control accelerated gastrointestinal motility induced by lipopolysaccharide—a bacterial product that is causally implicated in chemotherapy-induced diarrhoea.

In addition to its effect on gastrointestinal motility, which has clear applications in controlling chemotherapy-induced diarrhoea, the ECS exerts potent immunomodulatory effects in the gastrointestinal tract controlling intestinal inflammation [41]. Both synthetic CB receptor agonists and endocannabinoids have been shown to impair cellular and humoral immunity by reducing inflammatory cell recruitment, inducing T-cell apoptosis and suppressing the production of numerous pro-inflammatory cytokines and chemokines (e.g., TNF-α, IL-1β, IL-2, IL-6, IL-17, IFN-γ, CCL2 or CXCL10) [41, 68]. Therapeutically, both exogenous administration of cannabis and preventing endocannabinoid degradation by inhibiting FAAH have been shown to reduce colitis [60–63, 69]. In fact, FAAH inhibition and CB receptor activation have shown efficacy in mouse models of colitis and FAAH knockout mice are less susceptible to experimentally induced colitis compared to wild-type mice [69]. The ability of endocannabinoids (or exogenous cannabis products) to accelerate wound healing in the gut points to their ability to promote intestinal/mucosal barrier function, that is, the bonding of intestinal epithelial cells to create a uniform and restrictive barrier. This mechanism has been confirmed in vitro, with cannabinoids improving or maintaining paracellular permeability (i.e., leakiness) and tight junction protein expression in Caco-2 cells treated with *Clostridium difficile* toxin A and other barrier-directed insults (e.g., cytokines, EDTA) [41, 70]. These mucoprotective effects have also been reported preclinically, with cannabinoids reported to decrease intestinal permeability (and increase regulatory T-cell recruitment) in experimental colitis induced by dextran sulfate sodium (DSS) [41]. It has also been suggested that cannabinoids can modulate secretory processes in the intestinal epithelium which, when dysregulated, lead to altered osmotic forces and potentially diarrhoea [71–74].

An emerging area of interest with respect to the gastrointestinal microenvironment is the interaction between the ECS, cannabis and the gut microbiota [43, 75, 76]. The gut microbiota is a collection of micro-organisms (bacteria, viruses and fungi) that reside in the gastrointestinal lumen and mucosal niches, regulating host physiology and immune function. Importantly, these beneficial host-directed effects are best achieved when there is high microbial diversity and enrichment for commensal microbes. Chemotherapy indirectly impacts the diversity and composition of the gut microbiota, through the destruction of their mucosal niches and oxidative stress [77]. As such, a highly dysbiotic microbiota is a hallmark trait of chemotherapy, and an event documented to drive a range of adverse effects including fever, infection, diarrhoea, cachexia, weight loss, anxiety, cognitive impairment, cardiotoxicity and fatigue. Although a relatively new concept, emerging data suggests an interaction between the ECS and the gut microbiota. Most recently, cannabis extracts (CN1, CN2, CN6) were shown to increase microbial diversity and richness in a mouse model of metabolic disease whilst promoting enrichment of microbial taxa associated with health [78]. Further suggesting an ECS-microbiota interaction is the finding that germ-free mice (mice without a microbiota) are deficient in a number of ECS components, including the CNR1 [79]. A microbial taxa of particular interest is *Akkermansia muciniphila*, a mucus-degrading microbe implicated in gut inflammation [80] and chemotherapy side effects [81, 82]. This microbe is reportedly elevated in response to CNR1 antagonism with the compound SR141716A, although this was only demonstrated in obese mice [76, 83]. Despite this emerging evidence, it is unclear if medicinal cannabis influences the gut microbiota and if this mechanism underpins/delivers meaningful impacts for the host.

Collectively, this body of evidence strongly demonstrates the profound control that the ECS exerts on gastrointestinal function, regulating motility, barrier function and repair, immune function, secretion and potentially the microbiota. These data underscore the potential mucoprotective effects of exogenous cannabinoid administration or augmentation of the ECS. In the context of cancer care, this therefore supports strategies targeting ECS (via direct cannabinoid administration or inhibition of degradation) to control mucositis and promote a more resilient gastrointestinal microenvironment. Despite the scientific strength of this rationale, and the prevalence of mucositis (occurs in ~60% of patients treated with standard chemotherapy), there have been few attempts to explore the mucoprotective properties of medicinal cannabis in cancer care [55]. This may reflect the complexities of using/investigating medicinal cannabis during active cancer treatment.

## Using medicinal cannabis during active cancer treatment: precautions, challenges and potential benefits

To minimise both the depth and duration of mucositis, supporting the gastrointestinal microenvironment and controlling the constellation of symptoms with which mucositis is associated, medicinal cannabis should be used during active chemotherapy treatment. Of course, this raises some concerns regarding the possibility of adverse drug interactions with anti-cancer therapies and potential loss of anti-tumour efficacy. There has been limited investigation of how cannabis influences the anti-tumour efficacy of cancer treatment. Cannabis has been investigated for its effect on the pharmacokinetics of irinotecan and docetaxel, with no effects observed [84]. On the other hand, two recently reported observational studies indicate a negative impact of cannabinoids on immune checkpoint inhibitors (ICI) related cancer outcomes [85, 86]. Bar-Sela et al. highlighted in a prospective study that the concomitant use of prescribed cannabis was associated with lower response rates (39% vs 59%), median time to progression (3.4 months vs 13.1 months) and median overall survival (6.4 months vs 28.5 months) when compared to ICI therapy alone [85]. Another retrospective observational study from the same research group reported inferior outcomes for cannabinoid use along with nivolumab (an ICI) when compared to nivolumab among selected solid cancers [86]. While some evidence suggests that cannabis may impair the anti-tumour efficacy of immunotherapy [87, 88], recent evidence suggests cannabis may actually have a synergistic effect with immunotherapy. This has been shown both preclinically and clinically, with median survival in CT26 tumour-bearing mice treated with THC and an anti-PD1 antibody having significantly higher overall survival compared to controls [89]. Authors also reported higher overall survival (numerical, failed to reach statistical significance) in 201 people with non-small cell lung cancer undergoing monotherapy with pembrolizumab who used medicinal cannabis. Although this does not confirm a synergistic effect, it does indicate no detrimental effect. The only study in which a definite synergy has been identified was preclinical, where a combination of cannabigerol and anti-PD-1 resulted in enhanced tumour clearance and increased survival compared to monotherapy in tumour-bearing mice [90]. Given the evidence from low quality clinical trials, well conducted trials are required to assess the efficacy of cannabinoids as anti-cancer therapeutics either alone or in combination with other systemic cancer therapies.

In the context of chemotherapy, although there is no concrete evidence that suggests cannabis may impair its anti-tumour efficacy, given the increasing evidence for immune-mediated mechanisms enhancing chemoefficacy [91], this risk cannot be ignored and should be appropriately built into studies investigating medicinal cannabis in combination with standard chemotherapy. Although it remains exclusively experimental and prone to inflation, there is a growing body of evidence that suggests medicinal cannabis may in fact be a beneficial adjunct to standard chemotherapy, capable of inducing cell death or controlling proliferation by inducing endoplasmic reticulum (ER) stress [92, 93], proteosome inhibition [94], upregulation of matrix metalloproteinases [95] and reactive oxygen species activation [96]. In line with these findings, several studies have outlined the potential for cannabinoids to be used as anti-cancer agents either on their own or in combination with other systemic cancer therapies or radiotherapy [97, 98]. A recent review summarised the anti-cancer effects of cannabinoids to be mediated through multiple pathways including anti-proliferative, pro-apoptotic, pro-autophagy, anti-invasion and metastasis, anti-angiogenesis, and immunomodulation [99]. However, it is important to note that the majority of these findings have only been explored in vitro or in preclinical (animal) models and are subject to inflation in the public domain. As a result, these benefits have not been robustly translated into the clinical setting.

Three human clinical trials reported the results on the role of cannabinoids on patients with recurrent glioma [100–102]. Guzman et al. demonstrated that only two of nine patients with recurrent Glioblastoma (GBM) had reduced tumour proliferation when treated with intracranial Δ^9^-THC alone as monotherapy [100]. Another trial that combined Nabiximols (a mixture of plant-derived THC, CBD and non-cannabinoid compounds) with temozolomide (*N* = 27) resulted in a numerically higher 2-year overall survival (50% vs 22%, *P* = 0.13) when compared to placebo/TMZ [101]. Schloss et al. compared two different doses of THC/CBD combination as adjunct to standard treatment of recurrent high-grade gliomas (*N* = 88) and demonstrated an improved quality of life and an imaging-assessed tumour response in 11% while 34% had stable disease when compared to historical controls [102]. These clinical trials highlight that a small proportion of people with recurrent GBM may benefit from cannabinoids, however, there are no available predictive biomarkers that may identify responders and non-responders.

In addition to the impact on anti-tumour efficacy, the other main risk associated with medicinal cannabis use in parallel to active treatment is drug–drug interactions. Given the predominant role of MC for pain control in cancer care, its interaction with other analgesics is of interest. Evidence suggests that cannabis may in fact enhance opioid-induced analgesia, with synergistic analgesia observed when opioid/cannabinoid ligands are co-administered. In animal studies, either morphine or codeine produces synergistic antinociception when combined with THC [103–106], and similar synergies have been documented in humans [107]. However, these benefits must be taken in light of evidence that suggests this combination may increase tolerance to both forms of analgesia and increase the risk of “drug-liking” effects [88, 108]. There is also evidence to suggest drug–drug interactions between CBD and the non-steroidal anti-inflammatory, naproxen, although this is limited to in vitro evidence [109]. However, both CBD and THC are metabolised by CYP2C9, suggesting the possibility of impaired drug clearance [109] or renal/liver toxicity. However, there are no data to suggest CBD and THC cause renal/liver toxicity, and in fact there is data to suggest hepatoprotective effects [110] and prevention of cisplatin-induced renal toxicity [111]. However safety profiles of MC in the context of cancer care remains superficially addressed, underscoring the importance of regular renal and liver function tests, appropriately titrated dosing, and studies with co-primary endpoints that address efficacy and safety [88]. These studies should also endeavour to capture the patient experience with respect to milder adverse events such as dry mouth and fatigue/somnolence, given their association with both cancer therapy and MC.

While medicinal cannabis use during active chemotherapy presents some challenges, if approached carefully, there are a number of benefits that can be achieved. These benefits largely relate to the ability to control multiple symptoms early in their aetiology, rather than therapeutically targeting single symptoms that may have developed and persisted well after treatment ends. This holistic approach to controlling multiple symptoms is in line with recent suggestions by the MASCC, and also aligns with the scientific evidence that underpins the clinical phenomenon of symptom clustering [112–114].

**Fig. 2:**
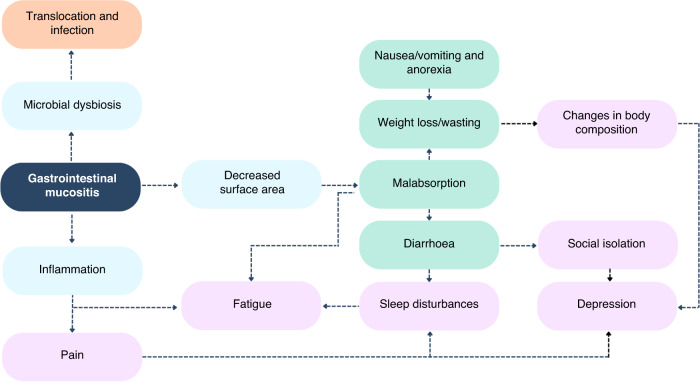
The centrality of gastrointestinal mucositis to infectious (orange), gastrointestinal (green) and neuropsychological (purple) symptoms commonly reported in people with advanced cancer undergoing chemotherapy. This positions gastrointestinal mucositis as an ideal therapeutic target.

We have outlined a clear rationale for how medicinal cannabis products, or augmentation of the ECS, modulates gastrointestinal function and exerts mucoprotective effects. It is therefore well positioned to target numerous mechanisms known to be involved in mucositis pathobiology and symptomatology. Given the documented role of mucositis and associated changes in the gastrointestinal microenvironment (e.g. gut microbiota dysbiosis) driving a range of intestinal and extraintestinal symptoms, medicinal cannabis in the active stages of chemotherapy may deliver broad-reaching benefits. This is particularly compelling when considering that medicinal cannabis, and the ECS, will likely have paralleled effects on these associated symptoms. For example, mucositis can cause taste changes and pain, leading to reduced oral intake (anorexia) and therefore clinically impactful weight loss. As such, targeting mucositis with cannabis, whilst simultaneously promoting food enjoyment and behaviours, will likely deliver meaningful impacts of weight maintenance and nutritional status. Similarly, diarrhoea and pain due to mucositis is anecdotally thought to cause sleep disturbances [115]. Again, by addressing a biological cause and providing symptomatic relief, the potential for meaningful impacts on patient quality of life is enhanced. This same framework can be applied to numerous, interacting consequences of mucositis and associated symptoms (Fig. 2).

By approaching symptom management in this manner, the magnitude of benefit is likely to be larger, and thus the health and well-being of people undergoing chemotherapy better maintained. This will deliver knock-on effects to patients, ensuring they remain in the workforce, thus reducing personal financial toxicity, and remain willing/capable of receiving their intended chemotherapy dosing ensuring optimal tumour response and progression-free/overall survival. This indirect effect on treatment efficacy is significant and should not be disregarded. For example, in a recent review of 874 women with advanced breast cancer, chemotherapy dose reductions of >15% significantly increased the risk of mortality [116]. Similarly, a reduction in total cumulative dose of neoadjuvant FEC-D (where it is <85% of intended dosing) decreased the length of survival in women with breast cancer [117]. Similar effects have been reported in ovarian cancer and colon cancer [118–120].

When considering the causes of dose reductions or modifications, adverse effects (i.e., side effects) are the most common cause, accounting for 82% of all dose reductions in a recent audit of 584 people undergoing adjuvant chemotherapy for stage III colon cancer [119]. As such, the provision of proactive supportive care that tackles symptom clusters at multiple points in their aetiology and progression is critical. In fact, the provision of supportive care early in symptom aetiology is recommended, with evidence illustrating both quality of life and survival benefits when this approach is adopted [121]. Furthermore, by addressing multiple symptoms concurrently, or targeting common underlying mechanisms of multiple symptoms, polypharmacy (5+ medications) can be reduced. More than 80% of people with advanced care report polypharmacy [122]. This approach is fragmented and places substantial burden on the patient who must navigate multiple medications, increasing their risk of adverse drug interactions and medical misadventure [123]. With recent calls to address the fragmented approach to supportive cancer care and symptom control [124], the ability of MC to transcend multiple symptoms is compelling and advantageous.

In considering how MC can be used to deliver substantive impacts for people undergoing chemotherapy, a number of practical matters must be carefully considered. Firstly, there are a range of cannabis extracts that are available for consumption in a variety of formulations ranging from dry leaves/buds which can be smoked or vaporised, to highly purified and processed isolates [125, 126]. Typically, the more readily accessible cannabis products are in the community, the lower the degree of purification and quality assurance. In light of high rates of self-reported use [16, 46, 48], this underscores the need to deliver evidence that (if shown to be beneficial) will ensure patients can access MC in more appropriate and safe formulations.

Cannabis is commonly available as an oil, which contains or is enriched for CBD and THC, typically in combination with many other cannabinoids and phytochemicals (e.g., terpenes) at varying proportions. Oils are a convenient method of administration and can be directly administered to the oral cavity for rapid mucosal absorption, however, they do require a degree of dexterity and are prone to inaccurate dosing as people typically administer their dose as a number of “drops” [88]. Similarly, sublingual or oral–mucosal sprays can be used. While direct application to the oral mucosa is a common and easy method of administration, it is important to consider how factors like oral mucositis may influence tolerance, as some sprays are prepared in an ethanol diluent which would be painful to apply to an ulcerated oral cavity. Similarly, it is unclear how oral mucositis impacts the rate of absorption. Oils can also be encapsulated for ingestion; however, this method of administration must be considered in the context of dysphagia and nausea/vomiting, which may impact the patient’s ability to swallow a capsule, or intestinal mucositis which may also influence the rate of absorption [127]. This underscores the need to conduct appropriate pharmacokinetic studies to understand how these unique factors associated with cancer, in particular active chemotherapy, impact the uptake and efficacy of cannabis. Further to this, there is evidence that suggests the presence of high-fat food impacts the bioavailability of cannabis, and thus this should be carefully considered in the design of capsules and other formulation strategies [32, 128]. Importantly, these methods of administration result in different clinical effects, particularly with respect to the timing and duration of the response [88]. These should be considered when selecting the time of administration (i.e., time of day) and the symptom or side effect(s) of interest.

In addition to the method of administration, the selection of specific cannabinoids and their relevant doses is critical. This decision needs to be guided by the specific symptom or side effect(s) of interest, with a clear scientific rationale for their use. When considering the MC intervention of interest, it is also important to acknowledge and respect the complexity of medicinal cannabis as an entire entity (i.e., a whole plant) in which the combination of numerous active compounds work cooperatively to elicit benefit. As such, while using synthesised isolates may be attractive from a pharmacological perspective, evidence suggests that the synergy of numerous cannabis compounds, a process referred to as the “entourage effect”, outweighs the benefits that can be gained from a single isolate or molecule.

In the context of mucositis and its constellation of symptoms, the use of CBD and THC (with other cannabinoids and compounds such as terpenes) is likely to be best positioned to deliver meaningful benefits based on their unique yet synergistic effects [13, 129]. Of note, CBD is documented to counteract the *undesirable* psychotropic effects of THC [130] and may therefore improve adherence. This synergy is in addition to the ability of these compounds to address different but related symptoms. For example, CBD is hypothesised to control self-perpetuating inflammatory pathways that ultimately dictate the depth and duration of mucosal injury to deliver clinically meaningful benefits [62, 131–133]. In parallel, THC has the capacity to provide complementary effects to control anxiety, promote food intake/appetite and sleep quality [134–137]. However, it cannot be ignored that while THC provides potential benefits for the patient, it certainly introduces additional medico-legal complexity, with many countries enforcing strict no-tolerance laws with respect to operating heavy machinery or driving motor vehicles. This may negatively impact patient well-being by impacting their employment prospects, or ability to live independently. It also poses challenges for patients that may have caring duties. Further advice regarding dosing has been summarised by Cyr and colleagues [88].

## The future of medicinal cannabis

The field of MC has been and continues to be difficult to navigate, reflecting the legislative challenges, variations in formulations and complexities of this emerging pharmacotherapy [33, 34, 38, 138]. To understand how to appropriately use this plant and its individual components in a therapeutic manner and avoid toxicity, well-designed in vitro and in vivo clinical pharmacology studies are required, particularly due to individual differences [138, 139]. Understanding the clinical pharmacology involved with MC will take the ‘guesswork’ out of the current largely uncontrolled and uncertain approach, which could provide the long wanting ‘safeguard’ for many vulnerable [140] individuals who are not benefiting from current practices and already suffering from poor quality of life due to undergoing cytotoxic chemotherapy and/or many other unpleasant conditions.

It is well-known that there are many cannabis species producing hundreds of compounds of which more than 100 chemicals are known as phytocannabinoids, with THC, CBD, terpenes and flavonoids being mostly abundant [31, 34]. Therefore, in order to provide this ‘safeguard’ with MC therapy, there are two key elements that need to be addressed. The first being, understanding the molecular mechanisms behind both the therapeutic and adverse effects of the various compounds within MC, and the second being, understanding the pharmacogenomics, pharmacokinetics and pharmacodynamics of the various products and formulations between individuals [34, 38, 139].

To achieve this, well-established genetic approaches should be adopted in the field of MC to facilitate precision medicine (‘selecting right drug’) [38, 139] and pharmacokinetic-guided approaches to facilitate precision dosing (‘selecting the right dose’) [31, 34, 141]. For the latter, specifically, exposure-response studies via therapeutic drug monitoring or pharmacogenomics may enable individualised therapy [38, 139], similar to how it has been done for anti-cancer therapeutics [142]. In addition, to provide true individualised or personalised dosing, population pharmacokinetic–pharmacodynamic (POP-PK/PD) models need to be developed to better understand how the population and individuals respond to these compounds, and which covariates (e.g., age, weight, gender, genotype, comedications, etc.) drive any variability in the pharmacokinetics and thus responses [140].

It has been well-documented that clinical trials are limited and of the available data, it is clear that varied design has resulted in the inability to compare and establish conclusive evidence. However, with personalised medicine currently in its peak in many research areas, such as antimicrobials, and anti-cancer drugs [143], it is an ideal time to build on current research and investigate which compounds should be isolated or combined in the appropriate formulations and administered to truly enable personalised MC therapy [34, 38, 139]. This will lead to a better understanding how exactly these compounds exert their effects and how this knowledge can be utilised to treat conditions such as mucositis which in turn has the potential to reduce symptom clustering and an array of treatments which cause additional challenges (polypharmacy, drug–drug interactions, adverse effects, poor quality of life and financial burden) for both the individuals and the healthcare systems. The need and opportunity to investigate how MC therapy can be personalised for individuals suffering from mucositis and other conditions is evident and brings on an exciting future for MC research.

While trial data remain inconclusive, it is likely that individual factors that dictate tolerance and efficacy have contributed to the variable and often contradictory results observed. For example, previous cannabis use is associated with lower anxiety after THC. Similarly genomic factors have been shown to predict the efficacy and tolerability of CBD. In a recent genomic study of patients with treatment-resistant epilepsy, single-nucleotide polymorphisms in certain genes were identified which were associated with a lower response and greater side effects of CBD [141]. The study also revealed genetic variants that were related to the likelihood of CBD-associated diarrhoea [141]. These findings present an opportunity for personalised pharmacogenomics-guided strategies for precise MC treatment that could be particularly advantageous for patients undergoing chemotherapy, already at risk of gastrointestinal side effects such as diarrhoea [141]. Understanding these factors will be critical in optimising the safe and effective use of cannabis in medical practice.

## Conclusions

Soon after the discovery of its chemical structure and ability to obtain various compounds from the plant in the late 1900s, as well as the description of the cannabinoid receptors and the endocannabinoid system in the 1990s, cannabis use for medical purposes has increased significantly with a steep rise in the last few years [31, 144]. It is evident that the majority of this use results from illegal access, however, this has been recognised and laws are in a fast-changing phase with several products approved and several others on a registered unapproved list. This provides new opportunities for users and prescribers to access MC products in a legal manner. In cancer care, self-reported cannabis use is prevalent, however, the evidence base is lacking due to inconsistencies in study design and outcomes. Moving forward, it is critical that research efforts integrate appropriate pharmacokinetic and mechanistic sub-studies to understand cannabis biology in the context of cancer and investigate its efficacy in a more holistic sense by considering its impact on clusters of related symptoms. In the context of mucositis, this is a compelling approach given the numerous symptoms that occur secondary to mucosal barrier injury and the already documented benefits medicinal cannabis has on gastrointestinal physiology, inflammation, and dysfunction.

## Data Availability

There is no other relevant data from this manuscript.
